# The Synthesis and Role of β-Alanine in Plants

**DOI:** 10.3389/fpls.2019.00921

**Published:** 2019-07-18

**Authors:** Anutthaman Parthasarathy, Michael A. Savka, André O. Hudson

**Affiliations:** The Thomas H. Gosnell School of Life Sciences, College of Science, Rochester Institute of Technology, Rochester, NY, United States

**Keywords:** β-alanine, L-alanine, non-proteinogenic, amino acid, secondary metabolites

## Abstract

Most studies on amino acids are focused on the proteinogenic amino acids given their essential roles in protein synthesis among other pathways. In addition to 20 ubiquitous amino acids used in protein synthesis, plants synthesize over 250 non-proteinogenic amino acids that are involved in the synthesis of compounds that are anti-herbivory, anti-microbial, response to abiotic stresses, nitrogen storage, toxins against both vertebrates/invertebrates, and plant hormones among others. One such non-proteinogenic acid is β-alanine, which is known mainly for studies on humans. β-Alanine forms a part of pantothenate (vitamin B5), which is incorporated into the universal carbon shuttling compounds Coenzyme A and acyl carrier protein, in all organisms including plants. The focus of this review, however, is on the biosynthesis, metabolism, and the role of β-alanine in plants. There are several functions of β-alanine unique to plants. It is accumulated as a generic stress response molecule involved in protecting plants from temperature extremes, hypoxia, drought, heavy metal shock, and some biotic stresses. There is evidence of its participation in lignin biosynthesis and ethylene production in some species. It is further converted to the osmoprotective compound β-alanine betaine in some species and converted to the antioxidant homoglutathione in others. The polyamines spermine/spermidine, propionate and uracil have been shown to be precursors of β-alanine in plants. However, plants vary in terms of their biosynthetic pathways, and the primary metabolism of β-alanine is far from settled.

## Introduction

**Figure 1:**
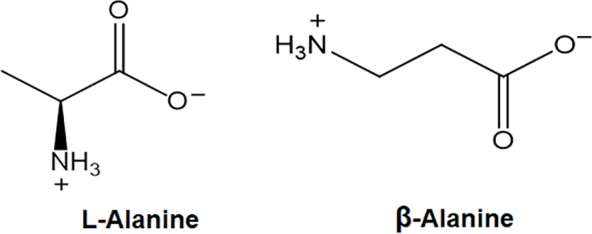
The chemical structures of L-alanine and β-alanine.

β-Alanine is a non-proteinogenic amino acid, where the amino group is at the β-position from the carboxylate group (IUPAC name = 3-aminopropanoic acid). In contrast to L-alanine, which is a proteinogenic amino acid, β-alanine has no stereo center (Figure 1). β-Alanine is incorporated into pantothenate (Vitamin B5), and therefore, is a precursor of Coenzyme A (CoA) and acyl-carrier protein, which shuttle carbon within the cell (Voet et al., 2006). β-Alanine is a component of carnosine, a dipeptide concentrated in muscle and brain tissue, which underlies the wide use of β-alanine in humans as a strength enhancing supplement. The vast majority of scientific articles about β-alanine deal with the exercise supplement aspect, please see for example Blancquaert et al. (2017). Much less is known about the role of β-alanine in plants. The focus of this manuscript is the current state of knowledge on the metabolism and role of β-alanine in plants.

Apart from the universal importance of β-alanine (as the precursor of CoA) in the synthesis of phospholipids, synthesis and degradation of fatty acids, and the operation of the tricarboxylic acid cycle, plants also employ β-alanine in secondary metabolism, including lignin biosynthesis (Broeckling et al., 2005). Moreover, β-alanine is involved in multiple stress responses in plants. LeSvels of β-alanine have been shown to be elevated significantly following heat shock in *Vigna unguiculata* cell cultures (Mayer et al., 1990). Various biotic and abiotic stresses on *Medicago truncatula* cell cultures resulted in elevated β-alanine levels, suggesting changes in the metabolism of CoA and its thioesters, which are essential in secondary metabolism (Broeckling et al., 2005). Both drought and heat stress were found to induce increased β-alanine levels in the model plant *Arabidopsis thaliana* (Kaplan et al., 2004; Rizhsky et al., 2004). Metabolomics studies reveal that in response to cadmium ions (heavy metal stress); *A. thaliana* accumulates multiple compounds, including L-alanine, β-alanine, and the polyamine putrescine (Sun et al., 2010).

β-Alanine aminotransferases from plants have been known since at least the late 1960s (Stinson and Spencer, 1969a,b). Work on enzymes from *Phaseolus vulgaris* (wax bean) cotyledons showed that β-alanine could be converted into the well-known plant signaling molecule ethylene (Stinson and Spencer, 1969a,b). In some plants, β-alanine is additionally converted to β-alanine betaine, an important quaternary ammonium osmoprotective compound that is involved in tolerance to both high salt concentration and low oxygen (Hanson et al., 1991, 1994; van Dongen et al., 2009; Rocha et al., 2010a,b). A role for β-alanine in recovery from waterlogging was suggested in an earlier study employing a hydroponic system (Drakeford et al., 1985). *In vitro* protection against high temperature stress for the enzyme lactate dehydrogenase (LDH) is afforded by β-alanine (Mehta and Seidler, 2005).

In some leguminous plants, β-alanine forms a part of the thiol tripeptide homoglutathione, which is involved in protection against heavy metal toxicity and free radicals (reactive oxygen species) (Klapheck et al., 1988; Moran et al., 2000). For this reason, pathways involving β-alanine are considered attractive targets for the metabolic engineering of plants, potentially including crop plants in a world facing a growing human population and increasing environmental stresses due to climate change. Work that is more recent suggests that β-alanine metabolism in *A. thaliana* involved in a variety of pathways and roles such as nitrogen utilization efficiency, response to hypoxia, osmoprotection, vitamin B5 and CoA metabolism (Parthasarathy et al., 2019).

## The Synthesis of β-Alanine In Plants

The biosynthesis of β-alanine in plants may be initiated from at least four different precursors, namely (1) the polyamines spermine/spermidine, (2) the carboxylic acid propionate, (3) the nucleotide base uracil, and (4) the proteinogenic amino acid L-aspartate. Among these, the first three are thoroughly characterized, while the fourth has long been postulated without conclusive experimental evidence. The physiological implications of each pathway are discussed below.

### The Synthesis of β-Alanine in Plants: The Polyamine Pathway

**Figure 2:**
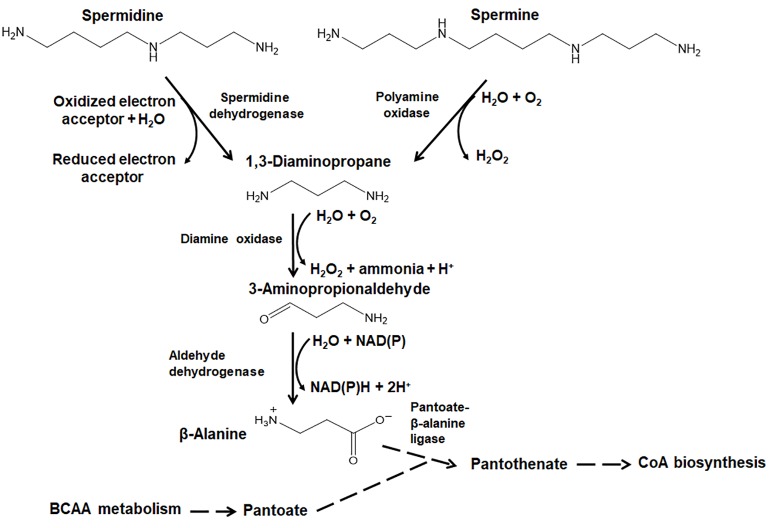
Anabolism of β-Alanine via the spermine and spermidine pathways. The enzyme class (EC) numbers shown in figure correspond to the following enzymes: spermidine dehydrogenase [EC 1.5.99.6], polyamine oxidase [EC 1.5.3.14], diamine oxidase [EC 1.4.3.22], aldehyde dehydrogenase [EC 1.2.1.3], and pantoate-β-alanine ligase (AMP-forming) [EC 6.3.2.1]. The dotted lines show the pantothenate and CoA biosynthesis pathway into which β-alanine can be fed. BCAA, branched chain amino acid.

The polyamines spermine and spermidine are converted *via* 1,3-diaminopropane to β-alanine (Figure 2) in many plants and demonstrated in maize shoots (Terano and Suzuki, 1978; Galston and Sawhney, 1990). Pericarp discs of tomato fruits were also shown to degrade spermidine to putrescine and β-alanine (Rastogi and Davies, 1989). Spermidine is cleaved by the FAD- and heme-containing enzyme spermidine dehydrogenase [EC 1.5.99.6] into 1,3-diaminopropane and 4-aminobutyraldehyde. Spermine degradation is facilitated by the incorporation of water and molecular oxygen by the enzyme polyamine oxidase [EC 1.5.3.14], yielding 1,3-diaminopropane, 4-aminobutyraldehyde, and hydrogen peroxide. The common intermediate 1,3-diaminopropane is then deaminated with the addition of water and molecular oxygen into 3-aminopropionaldehyde, hydrogen peroxide, and ammonia by the enzyme diamine oxidase [EC 1.4.3.22]. Finally, oxidation of 3-aminopropionaldehyde with the addition of water by the NAD(P)-dependent aldehyde dehydrogenase [EC 1.2.1.3] yields β-alanine. Spermidine oxidation *via* these enzymes is induced by abiotic stresses (Kamada-Nobusada et al., 2008; Moschou et al., 2008a,b).

*At*ALDH10A8, an NAD^+^-dependent aminoaldehyde dehydrogenase [EC 1.2.1.19] in *A. thaliana* was shown to convert 3-aminopropionaldehyde into β-alanine *in vitro*, and similar enzymes were studied in apple fruit (Zarei et al., 2015, 2016). However, unlike gamma-aminobutyric acid (GABA), which was specifically accumulated in response to salinity, β-alanine was not, suggesting that β-alanine is accumulated as part of a non-specific rather than a specific stress response (Zarei et al., 2016). *A. thaliana* has been shown to survive without the ability to make spermine, but spermidine synthesis is essential to survival. This suggests that spermidine (but not spermine) could be linked to pantothenate biosynthesis *via* β-alanine (Imai et al., 2004). The condensation of β-alanine with pantoate in plants (Figure 2) yields pantothenate (vitamin B5), which is essential for all organisms as a precursor to the 4′-phosphopantetheine moiety of coenzyme A (CoA) and acyl carrier protein (Webb et al., 2004; Webb and Smith, 2011).

As seen in Figure 2, there is an intriguing link between the metabolism of the branched chain amino acids (BCAA) and β-alanine. It appears that there is a feedback loop whereby a change in the levels of β-alanine may be effected *via* BCAA degradation. Since valine and isoleucine may serve as precursors for propionyl-CoA, recent studies in this regard are discussed in the next section.

### The Synthesis of β-Alanine in Plants: The Propionate Pathway

**Figure 3:**
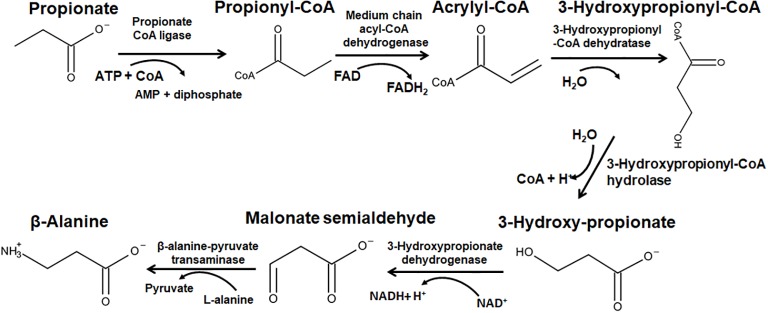
The anabolism of β-alanine *via* the propionate pathway. The enzymes shown in the figure correspond to the following EC numbers: propionate CoA ligase = [EC 6.2.1.17]; medium chain acyl-CoA dehydrogenase = [EC 1.3.8.7]; 3-hydroxypropionyl-CoA dehydratase = [EC 4.2.1.116]; 3-hydroxypropionyl-CoA hydrolase = [EC 3.1.2.4]; 3-hydroxypropionate dehydrogenase = [EC 1.1.1.59], and β-alanine-pyruvate transaminase = [EC 2.6.1.18].

Propionate may act as the precursor for β-alanine in the pathway involving a β-alanine aminotransferase (Figure 3). Propionate is activated with the hydrolysis of ATP to propionyl-CoA A by propionate CoA ligase [EC 6.2.1.17]. Propionyl-CoA is then oxidized to acrylyl-CoA by the FAD-dependent medium chain acyl-CoA dehydrogenase [EC 1.3.8.7]. 3-hydroxypropionyl-CoA dehydratase [EC 4.2.1.116], then hydrates acrylyl-CoA yielding 3-hydroxypropionyl-CoA. Hydrolysis of the CoA ester by 3-hydroxypropionyl-CoA hydrolase [EC 3.1.2.4] generates 3-hydroxyproiponate, which is further oxidized to malonate semialdehyde by the NAD-dependent 3-hydroxypropionate dehydrogenase [EC 1.1.1.59]. The final step in this pathway is the transamination catalyzed by the pydridoxal-5′-phosphate (PLP)-dependent β-alanine-pyruvate transaminase [EC 2.6.1.18], whereby an amino group is transferred from L-alanine to malonate semialdehyde, yielding β-alanine and pyruvate (Hayaishi et al., 1961; Stinson and Spencer, 1969a,b). After 2001 study identified a β-alanine aminotransferase in *A. thaliana* (Liepman and Olsen, 2001), it was hypothesized that this pathway may be involved in pantothenate production. In *A. thaliana*, there is a gene encoding for the pantothenate synthase (PtS) enzyme, which condenses pantoate with β-alanine to yield pantothenate (Genschel et al., 1999; Ottenhof et al., 2004). It should be noted that homologous gene/s encoding aspartate decarboxylase (ADC), the enzyme that generates β-alanine in the bacterial pantothenate pathway were not identified in the plant (Ottenhof et al., 2004).

The propionate pathway connects the metabolism of L-alanine and β-alanine in plants, since the final step requires the former amino acid as the amino donor (Figure 3). Therefore, a change in the levels of one or the other of the two amino acids may have wide-ranging physiological effects. Gene regulation studies of alanine aminotransferase has been studied in many plants in response to low-oxygen stress, light, and nitrogen, and it was demonstrated that hypoxia induced the expression of two distinct alanine aminotransferase genes in *A. thaliana* (Miyashita et al., 2007). The function of the gene product from the locus tag At3g08860 was unknown for a long time, but was recently shown to encode a β-alanine/L-alanine aminotransferase using an *in vivo* functional complementation approach (Parthasarathy et al., 2019). The same study reported that a definitive preference of the corresponding enzymatic activity toward the synthesis of β-alanine. The gene corresponding to the β-alanine/L-alanine aminotransferase was shown to be most highly expressed in *A. thaliana* roots (Schmid et al., 2005). The enrichment of the β-alanine aminotransferase enzyme in the roots suggests that β-alanine possibly has a specific protective function in the root tissue. Earlier, hydroponics studies in *Helianthus annuus L.* revealed that roots which were excised and flooded for 24 h had reduced uptake of radioactive β-alanine than non-flooded roots and also that they exuded more radioactive β-alanine into distilled water (Drakeford et al., 1985). This suggests, first, that there is a regulation of β-alanine uptake (if available in the medium), and second, that flooded roots may exude β-alanine for protective purposes. Therefore, it is likely that plants require enhanced expression of the β-alanine aminotransferase in the roots to facilitate a β-alanine based defense response when faced with hypoxia or flooding.

Additional studies have addressed the links between branched-chain amino acids and β-alanine, since valine and isoleucine degradation can produce propionyl-CoA. Using radiolabeled precursors of both isoleucine and valine, it was shown in *A. thaliana* that only isoleucine directly generated β-alanine. However, if seedlings were treated with valine, an increase in the levels of β-alanine resulted, suggesting an indirect effect *via* amino acid homeostasis (Perrett et al., 2017). Seedlings harboring a mutated methylmalonate semialdehyde dehydrogenase (MMSD, At2g14170) gene cannot convert valine into propionyl-CoA (since the Mmsd-1 protein catalyzes the final step leading to propionyl-CoA); but the equilibrium is shifted toward β-alanine, presumably by the amino acid homeostatic mechanism (Perrett et al., 2017). More recently, feeding of 13C-labeled isoleucine and propionate were shown to result in the production of C^13^-labeled 3-hydroxypropionate and β-alanine in *A. thaliana* seedlings, offering direct evidence for the metabolic link between isoleucine and β-alanine *via* propionyl-CoA (Goldfarb and Rouhier, 2019). Further studies in transgenic *A. thaliana* and wheat seedlings confirmed that isoleucine degradation could initiate β-alanine synthesis (Rouhier et al., 2019). This suggests that isoleucine may serve as an additional precursor for β-alanine in plants (apart from those known already) and since valine is a precursor of pantoate, pantothenate production could depend on BCAA catabolism.

### The Synthesis of β-Alanine in Plants: The Uracil Pathway

**Figure 4:**
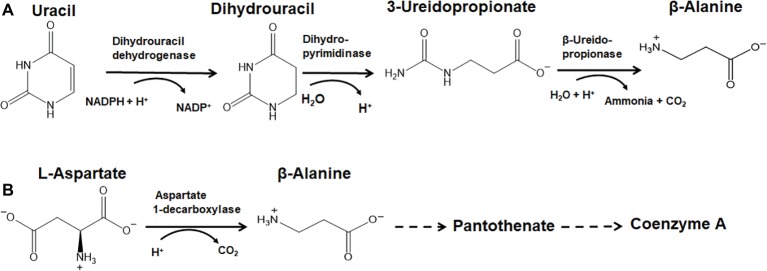
The anabolism of β-Alanine biosynthesis *via* uracil and L-aspartate. **(A)** The nucleotide base uracil can be degraded to β-alanine. The enzymes in this pathway are dihydrouracil dehydrogenase [EC 1.3.1.2], dihydropyrimidinase [EC 3.5.2.2] and β-ureidopropionase [EC 3.5.1.6]. **(B)** Aspartate 1-decarboxylase [EC 4.1.1.11] can directly decarboxylate L-aspartate into β-alanine, which may feed into Coenzyme A biosynthesis *via* pantothenate.

The nucleotide base uracil is reduced by NADPH-dependent dihydrouracil dehydrogenase [EC 1.3.1.2] into dihydrouracil (Figure 4A); in *A. thaliana,* this gene is identified with the At3g17810 locus. The hydrolysis of dihydrouracil into 3-ureidopropionate is catalyzed by dihydropyrimidinase [EC 3.5.2.2] (Figure 4A); the accession IDs in *A. thaliana* are At5g12200 and At5g12200.1. Further hydrolysis by β-ureidopropionase [EC 3.5.1.6] eliminates ammonia and carbon dioxide, yielding β-alanine as shown in Figure 4A (Campbell, 1960; Traut and Loechel, 1984); the *A. thaliana* accession IDs are At5g64370 and At5g64370.1. The degradation of uracil and thymine to produce carbon dioxide, ammonia, β-alanine, and γ-aminoisobutyrate has been shown to occur in seedlings of *Brassica napus* (rapeseed), while the β-ureidopropionase enzyme of maize was characterized by its overexpression in *Escherichia coli* (Tsai and Axelrod, 1965; Walsh et al., 2001). The existence of this pathway in pine trees was inferred already in the 1960s (Barnes and Naylor, 1962). In the halotolerant members of the leadwort or plumbago family (*Plumbaginaceae*) of perennial plants, uracil is only one of many sources for β-alanine (Duhazé et al., 2003). Thus, the distribution of this pathway in higher plants varies widely.

### The Synthesis of β-Alanine in Plants: The Aspartate Pathway

L-aspartate may be decarboxylated by aspartate 1-decarboxylase [EC 4.1.1.11] also yielding β-alanine, which may enter Coenzyme A biosynthesis (Figure 4B). This enzyme abbreviated ADC and encoded by the *panD* gene has been well characterized in prokaryotes (Williamson and Brown, 1979; Merkel and Nichols, 1996). However, its presence in plants has never been identified and or confirmed. Bioinformatics analysis based on sequence homology using the FUGUE tool failed to find homologs in either *A. thaliana* or the yeast *Saccharomyces cerevisiae* genomes, casting doubt on whether this pathway crossed the divide between prokaryotes and eukaryotes (Shi et al., 2001; Ottenhof et al., 2004). With the availability of numerous genome sequences of plants and fungi available since 2004, further inquiry of *panD* orthologs using bioinformatics tools is warranted.

Since only plants and microbes synthesize pantothenate *de novo*, there is significant interest in engineering this pathway in plants in order to fortify food crops with pantothenate, which is essential for animals since they lack the enzymes required to biosynthesize the compound (Coxon et al., 2005; Chakauya et al., 2008). Also, since only plants and microbes contain the pathway for pantothenate, it is also considered an attractive target for herbicides, fungicides and antibiotic development (Coxon et al., 2005). Increased heat tolerance *via* overproduction of β-alanine and pantothenate content could be achieved in transgenic tobacco plants harboring the gene for the *E. coli* ADC enzyme (Fouad and Rathinasabapathi, 2006). Later, the same system was also shown to enhance photosynthesis and to augment biomass production in response to higher temperatures (Fouad and Altpeter, 2009). These are important practical advances that could pave the way for heat resistant transgenic food crops that are better suited in warmer climate zones and in changing climates, where loss in agricultural productivity with increasing temperatures might be a concern.

## The Utilization of β-Alanine in Other Plant Pathways

### β-Alanine Betaine

**Figure 5:**
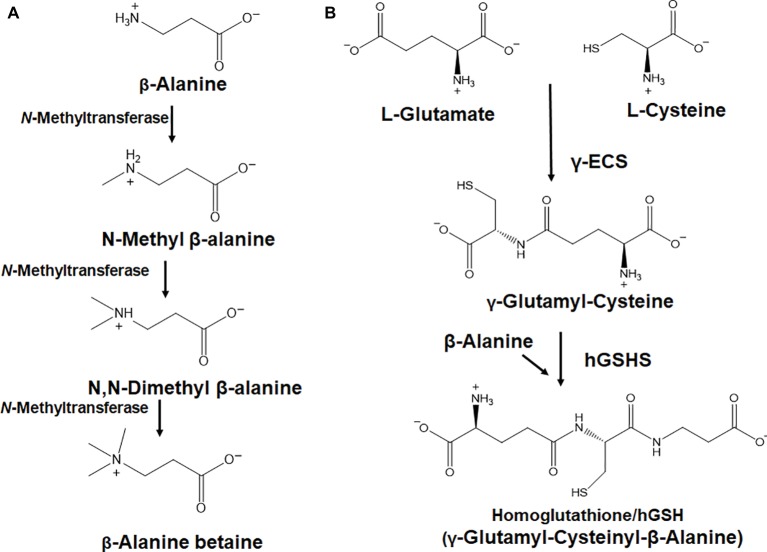
The conversion of β-alanine to important secondary metabolites. **(A)** A trifunctional, *S*-adenosyl-L-methionine (SAM)-dependent *N*-methyltransferase [EC 2.1.1.49], performs iterative *N*-methylation of β-alanine to β-alanine betaine. **(B)** Homoglutathione biosynthesis involves the enzymes, glutamate cysteine ligase (GCL) or γ-ECS (γ-glutamyl-cysteine synthetase) [EC 6.3.2.2] and a β-alanine specific homoglutathione synthase (hGSHS) [EC 6.3.2.23], both of which perform specific amino acid condensations at the expense of ATP.

β-Alanine betaine is an osmoprotective compound accumulated by most members of the highly stress-tolerant leadwort or plumbago family (*Plumbaginaceae*). β-Alanine betaine is synthesized by S-adenosyl-*L*-methionine (SAM)-dependent enzymatic *N*-methylation of β-alanine (Figure 5A) *via N*-methyl β-alanine and *N,N*-dimethyl β-alanine (Raman and Rathinasabapathi, 2003). Most members of the highly stress-tolerant *Plumbaginaceae* accumulate β-alanine betaine instead of glycine betaine (Hanson et al., 1991, 1994). It was proposed that β-alanine betaine is a more suitable osmoprotectant than glycine betaine under saline hypoxic conditions because the first step in glycine betaine synthesis catalyzed by choline monooxygenase [EC 1.14.15.7] requires molecular oxygen (Hanson et al., 1991, 1994). Further, β-alanine betaine accumulation was proposed to be an evolutionary strategy to avoid the metabolic limitations existing for choline oxidation under hypoxic conditions, because β-alanine betaine is synthesized from the ubiquitous primary metabolite β-alanine (Hanson et al., 1994).

### Homoglutathione

In many legumes, the thiol tripeptide homoglutathione (hGSH; γGlu-Cys-βAla) (Figure 5B) can partially or fully replace the better-known thiol, glutathione. γ-Glu-Cys is formed from L-glutamate and L-cysteine by glutamate cysteine ligase (γ-Glutamylcysteine synthetase or γ-ECS) at the expense of ATP, and γ-Glu-Cys and β-alanine are condensed at the expense of ATP by a specific hGSH synthetase (hGSHS), an enzyme which has affinity for β-alanine and low affinity for glycine (Macnicol, 1987; Klapheck, 1988; Klapheck et al., 1988). Multiple cellular compartments are involved in the biosynthesis of hGSH and fractionation of the root nodules demonstrated that the bacteroids contain high hGSH concentrations and the highest specific activities of GSHS, suggesting a critical role in nitrogen fixation processes in the root nodules of legume plants (Moran et al., 2000). More recent work in *M. truncatula* also showed that hGSH is essential for the growth of parasitic nematode worms, which infect plant roots and force the differentiation of root cells into giant cells. In addition, it was also shown that hGSH-depleted roots did not suffer similar damage by nematodes (Baldacci-Cresp et al., 2012). Thus, hGSH may play key roles in plant defense and could be the target of future interventions into improving plant resistance to nematodes.

## Conclusions

Although underappreciated, the non-proteinogenic amino acid β-alanine has important roles in plant physiology and metabolism, directly as a defense compound that enables plants to withstand various stresses such as hypoxia, waterlogging and drought, and indirectly as a precursor to the compounds pantothenate and CoA, which are involved in a variety of functions. Furthermore, the amino acid is converted into β-alanine betaine, which has additional protective functions such as salt tolerance, and homoglutathione, which may be critical for nitrogen fixation.

## Author Contributions

All authors listed have made a substantial, direct and intellectual contribution to the work, and approved it for publication.

### Conflict of Interest Statement

The authors declare that the research was conducted in the absence of any commercial or financial relationships that could be construed as a potential conflict of interest.
